# Effect of patient-initiated versus fixed-interval telePRO-based outpatient follow-up: study protocol for a pragmatic randomised controlled study

**DOI:** 10.1186/s12913-017-2015-8

**Published:** 2017-01-26

**Authors:** Liv Marit Valen Schougaard, Caroline Trillingsgaard Mejdahl, Klaus Hvam Petersen, Anne Jessen, Annette de Thurah, Per Sidenius, Kirsten Lomborg, Niels Henrik Hjollund

**Affiliations:** 1 AmbuFlex, Regional Hospital West Jutland, Herning, Denmark; 2 The Research Programme in Patient involvement, Aarhus University Hospital, Aarhus, Denmark; 3 Department of Rheumatology, Aarhus University Hospital, Aarhus, Denmark; 4 Department of Clinical Medicine, Aarhus University, Aarhus, Denmark; 5 Department of Neurology, Aarhus University Hospital, Aarhus, Denmark; 6 Department of Public Health, Aarhus University, Aarhus, Denmark; 7 Department of Clinical Epidemiology, Aarhus University Hospital, Aarhus, Denmark

**Keywords:** Patient-reported outcomes, TelePRO, Clinical practice, Outpatient clinic, Outpatient follow-up, Open access, Randomised controlled trial

## Abstract

**Background:**

The traditional system of routine outpatient follow-up of chronic disease in secondary care may involve a waste of resources if patients are well. The use of patient-reported outcomes (PRO) could support more flexible, cost-saving follow-up activities. *AmbuFlex* is a PRO system used in outpatient follow-up in the Central Denmark Region. PRO questionnaires are sent to patients at fixed intervals. The clinicians use the PRO data to decide whether a patient needs a visit or not (standard telePRO). PRO may make patients become more involved in their own care pathway, which may improve their self-management. Better self-management may also be achieved by letting patients initiate contact. The aim of this study is to obtain data on the effects of patient-initiated follow-up (open access telePRO) on resource utilisation, quality of care, and the patient perspective.

**Methods:**

The study is a pragmatic, randomised, controlled trial in outpatients with epilepsy. Participants are randomly assigned to one of two follow-up activities: a) standard telePRO or b) open access telePRO. Inclusion criteria are age ≥ 15 years and previous referral to standard telePRO follow-up at Aarhus University Hospital, Denmark. Furthermore, patients must have answered the last questionnaire via the Internet. The number of contacts will be used as the primary outcome measure. Secondary outcome measures include well-being (WHO-5 Well-Being Index), general health, number of seizures, treatment side effects, mortality, health literacy (Health Literacy Questionnaire), self-efficacy (General Self-Efficacy scale), patient activation, confidence, safety, and satisfaction. In addition, the patient perspective will be explored by qualitative methods. Data will be collected at baseline and 18 month after randomisation. Inclusion of patients in the study started in January 2016. Statistical analysis will be performed on an intention-to-treat and per-protocol basis. For qualitative data, the interpretive description strategy will be used.

**Discussion:**

The benefits and possible drawbacks of the PRO-based open access approach will be evaluated. The present study will provide important knowledge to guide future telePRO interventions in relation to effect on resource utilisation, quality of care, and the patient perspective.

**Trial registration:**

ClinicalTrials.gov: NCT02673580 (Registration date January 28, 2016)

**Electronic supplementary material:**

The online version of this article (doi:10.1186/s12913-017-2015-8) contains supplementary material, which is available to authorized users.

## Background

The Danish health care system is changing from inpatient towards a greater outpatient activity. From 2002 to 2009, there was a 50% increase in outpatient activity in Denmark, primarily related to the number of contacts per patient [1]. At the same time, there appears to be a growing need of health care services especially for the growing group of patients with chronic diseases and an increased focus on patient involvement. The challenge is to manage this without compromise on quality of care and patient outcomes. Follow-up visits for patients with chronic diseases in secondary care are traditionally based on regular pre-booked visits, which may be arranged when the patient is well. Thus patients as well as clinicians may find such visits unnecessary. The volume of appointments leads to capacity issues in outpatient clinics that struggle to respond rapidly to patients’ requests for help [2].

One way of handling this challenge may be to let patients report essential information on health status and symptoms from home before or instead of visiting the outpatient clinic. Patients’ own reports on health condition are termed patient-reported outcomes (PRO). The American Food and Drug Agency definition of PRO, *“A measurement based on a report that comes directly from the patient about the status of a patient’s health condition without interpretation of the patient’s response by a clinician or anyone else”* [3], focuses on the source of information and points out the importance of the patient perspective. The use of PRO in clinical practice is becoming increasingly common, and studies have reported improved patient-clinician communication, more effective self-management, and better utilisation of resources when PROs are used, whereas findings related to effects on patient outcomes are less consistent [4–7].

PRO may facilitate patient involvement because the problems reported as important by the patient are taken into consideration in the decision-making process [8–10]. However, patient involvement is not a goal in itself but rather a means to increase the patient’s self-management. Self-management refers to the individual’s ability to manage symptoms, treatment, physical and psychosocial consequences, and life style changes inherent in life with a chronic disease [11]. In practice, PRO is supposed to promotes a patient-centred dialogue between the patient and the clinicians in which the patient’s view and opinion on his health are included. Thus, implementing PRO into clinical practice allows patients to actively participate in their own care, by which their self-management may improve [8].

*AmbuFlex* is a generic clinical PRO system which is not limited to specific patient groups, organisations or medical record systems [12]. As of December 2015, AmbuFlex had been implemented in nine patient groups at 15 outpatient clinics in Denmark [13]. An analysis initiated by the Danish government based on experiences with AmbuFlex has demonstrated a positive national business case and considerable quality gain [14]. The Danish government and Danish regions, who run the public hospitals, have decided on an agreement for nationwide implementation of PRO in three diagnostic group, including epilepsy, before 2020. AmbuFlex was implemented for epilepsy outpatients at Aarhus University Hospital in March 2012 and is now used at three neurological departments in the Central Denmark Region. As of August 2016, 4,513 epilepsy outpatients have been referred to AmbuFlex, which are about two-thirds of all epilepsy outpatients in the region. The PRO questionnaire used contains information on specific aspects of daily life with epilepsy and has been developed in close cooperation with clinicians and patients. Face validity is fundamental and has been ensured during the development of the questionnaire [12, 13]. A graphical PRO overview is presented to the clinicians, who use the PRO data for clinical decisions together with other available clinical data in the record to decide whether the patient needs a visit or not. If a PRO questionnaire is used to evaluate the patient’s need for a hospital visit, the PRO data must be obtained outside the hospital. This is called tele-patient-reported outcome (telePRO) [12]. Experiences from epilepsy outpatient clinics have shown that of 8,256 PRO-based contacts, 48% were handled without additional contact to the patient other than the PRO questionnaire [13]. A preliminary interview study has indicated that patients experience greater flexibility in care, the saving of time, improved communication with the clinicians, and increased knowledge about their own disease [13, 15].

The AmbuFlex method used at the three neurological departments is called *standard telePRO.* In standard telePRO, regular scheduled visits are replaced with fixed questionnaires at intervals similar to those of the former pre-booked visits. A patient-initiated approach “open access” telePRO has been developed in which patients have access to their own PRO data and are able to initiate contact with the clinic by filling in a PRO questionnaire. A review by Whear et al. investigated the effectiveness of patient-initiated clinics in chronic conditions in secondary care and included seven randomised trials. The review found that the risk of harm from using the patient-initiated clinic model is low in patients with breast cancer, inflammatory bowel disease, and rheumatoid arthritis. The included studies found few significant differences in clinical outcomes between traditional appointment scheduling and the patient-initiated follow-up method. In four of the studies, the patient-initiated model was associated with savings in clinician time and resource use [2]. A review by Taneja et al. that included five of the same randomised studies reached the same conclusion [16], while another review showed no significant differences in psychological and health-related quality of life outcomes between consultant-led and patient-initiated clinics. Patients have reported better satisfaction in patient-initiated clinics compared to usual care [17]. The patient-initiated method used was broadly the same in the studies included in the three reviews. Patients could request clinical advice by calling the clinic and, if necessary, arranging an appointment to see a clinician. However, none of the included studies used PRO as the main access point in the open access intervention, and all studies contain methodological limitations [2, 16, 17].

### Objectives

The aim of this study is to provide insight into the effects of patient-initiated telePRO follow-up. The specific aims are to compare resource utilisation, quality of care, and the patient perspective of two outpatient follow-up activities: a) standard telePRO (fixed-interval telePRO follow-up) and b) open access telePRO (patient-initiated telePRO follow-up). We hypothesise that 1. Number of contacts is less in open access telePRO, 2. Quality of care in open access telePRO is at least as good as in standard telePRO, and 3. Patient self-management and experiences in open access telePRO are better than standard telePRO.

## Methods

The study follows the (Additional file 1: SPIRIT checklist): Standard protocol items for clinical trials [18].

### Design

This study is a pragmatic two-arm randomised controlled trial. Participants are randomly assigned to one of two follow-up activities: (a) standard telePRO or (b) open access teleRPO.

### Study population

Participants are epilepsy outpatients recruited from the epilepsy clinic at Aarhus University Hospital in Central Denmark Region, Denmark.

Inclusion criteria

- Age ≥ 15 years
- Diagnosis or suspicion of epilepsy (IC-D 10 codes: G40, Z033a, DR568 and DR568E)
- Already referred to standard telePRO by a clinician
- Able to answer the questionnaire via the Internet, indicated by having answered the last questionnaire via the Internet

Exclusion criterion

- Referred to telePRO follow-up with proxy questionnaire. Patients can be referred to a proxy questionnaire if they have cognitive problems and need help from a relative or health professionals.

### Intervention

#### Reference group – standard telePRO

**Fig. 1:**
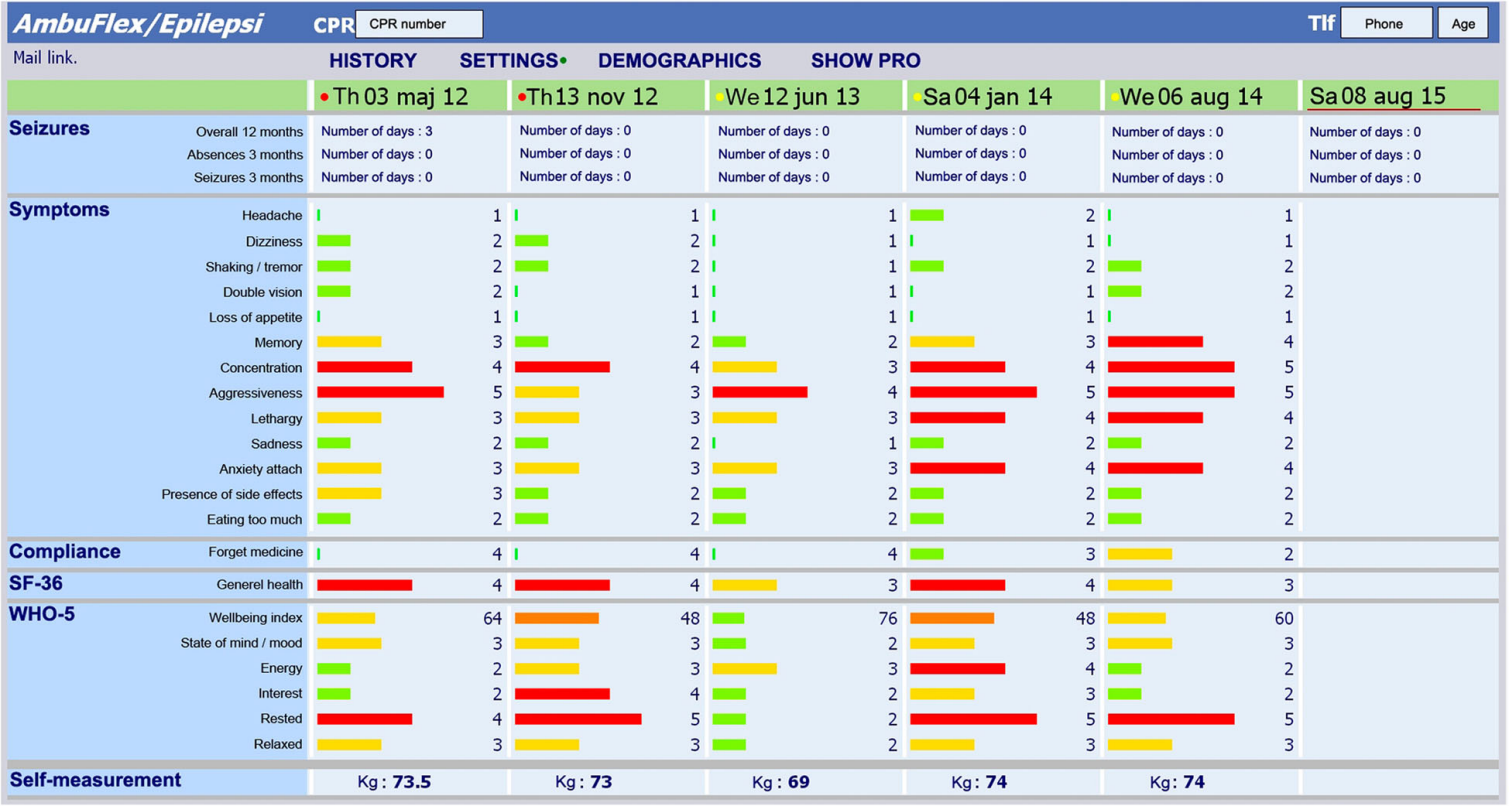
Screen capture of the clinicians’ overview in epilepsy clinics accessed from the Electronic Health Record of Central Denmark Region [13]. The colour dots in the upper row indicate the result of the automated PRO algorithm (red: definite need of contact, yellow: possible need of contact, green: no need of contact). Note that the colours of the bars have different meanings. The bars indicate the severity of the symptoms reported by the patient. A red or orange bar indicates a self-reported problem, a yellow bar some problem, and a green bar indicates no problems. Note: Labels were translated from Danish

AmbuFlex (standard telePRO) is used in three epilepsy outpatient clinics in Central Denmark Region. In standard telePRO, outpatient follow-up activity is determined by a clinician and patients receive a questionnaire at fixed intervals (3, 6, or 12 months). The questionnaire includes information about aspect of daily life with epilepsy such as seizures, symptoms, medication adherence, and social aspects. Responses are automatically processed according to a specific algorithm and given a “green”, “yellow”, or “red” status. A red status indicates that the patient needs or wishes contact with the clinic, a green status that the patient has no current need of attention, while a yellow status indicates that the patient may need to be seen in the clinic, but a clinician has to decide whether further contact is needed. The patient can always overrule a decision by requesting contact. They can choose two different contact forms in the questionnaire: telephone consultation or a face-to-face consultation at the clinic. Non-responders get three reminders and are contacted if do not respond. Clinicians keep track of incoming yellow and red responses, and non-responders, and this information is presented on a PRO alert list. The PRO overview (Fig. 1) is presented graphically to the clinician within the electronic health record system, and used as decision aid together with other available health record information to decide whether the patient needs a visit or not [13].

#### Intervention group – open access telePRO

In open access, contact to the outpatient clinic is initiated by the patient by filling in a PRO questionnaire. The same questionnaire is used as in standard telePRO, but the patient decides when to respond. The patients can access a PRO overview, “My Epilepsy”, customised for patient use via a secure login at the Danish national health website “Sundhed.dk”. The clinicians handle questionnaires in the same way as in standard telePRO.

#### The open access website “My Epilepsy”: design and features

**Fig. 2:**
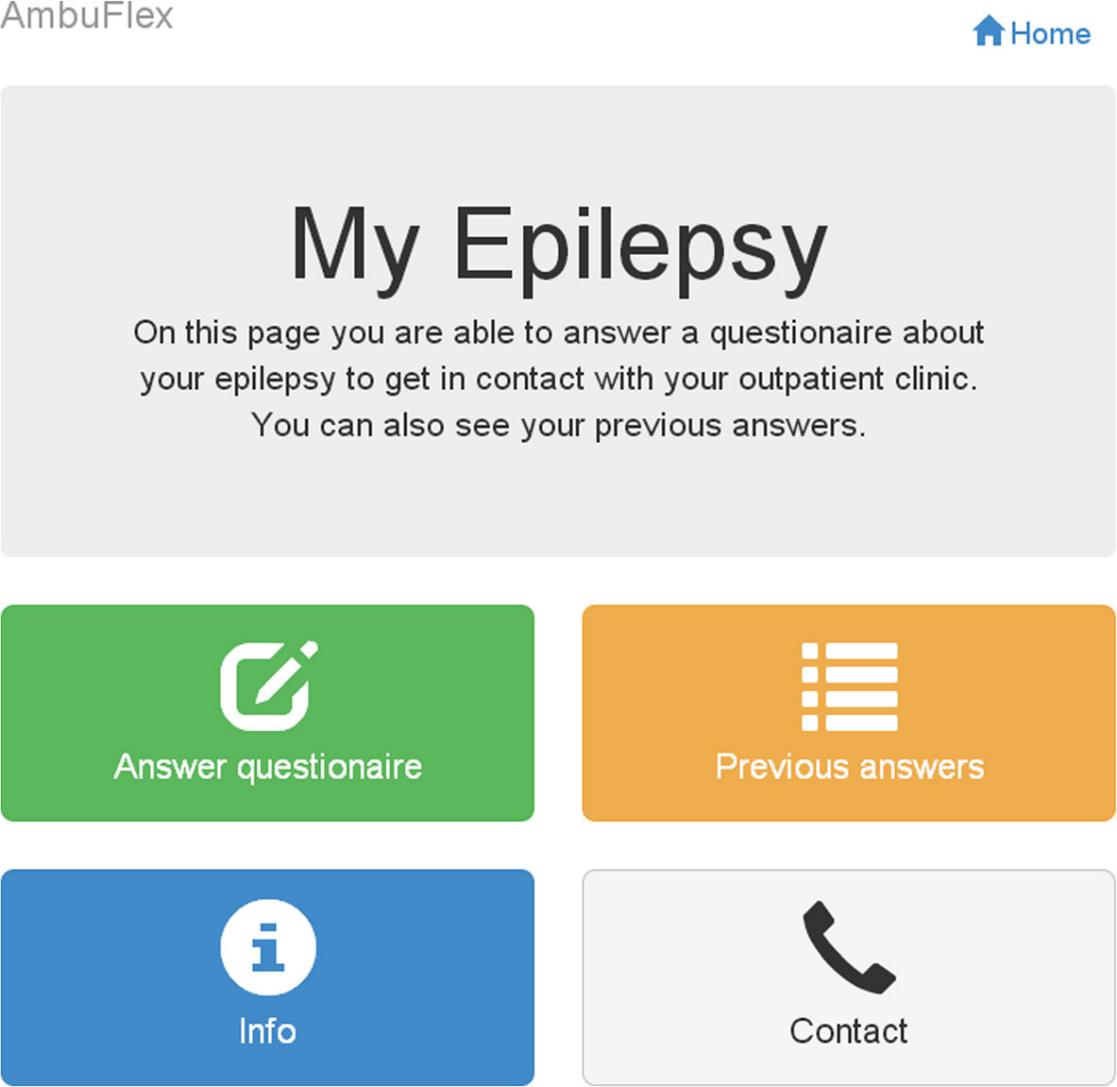
The open access telePRO website “My Epilepsy”. Note: Labels were translated from Danish

A prototype website, “My Epilepsy”, was developed to collect PRO in patient-initiated outpatient follow-up. The website was linked with the Danish National Health Website ‘Sundhed.dk’. The website, “My Epilepsy”, was customised for patient use and designed to allow patients to: a) answer a PRO questionnaire to get in contact with the clinic, b) view their personal PRO data (previously questionnaire responses), c) view information about the epilepsy questionnaire and specific questions, and d) have access to contact information to the epilepsy outpatient clinic. A research team that included, outpatients with epilepsy and experts in telePRO, patient involvement, software technology, clinical epilepsy, provided inputs to design the prototype website. The research team developed the initial website specifications, constructed the website, and elicited feedback from epilepsy outpatients (*n* = 6), using cognitive interviewing techniques to study the manner in which the patients understood and responded to the website. The interface is shown in Fig. 2. Patients emphasised the importance of a user-friendly interface with clear and concise information. Patients were interested in tracking change over time and in using the website because it gave them the potential to communicate with their clinicians at a time decided by themselves. They found it conceivable that access to their previous questionnaires could give them a better understanding of their chronic disease. Finally, they pointed out the need for a telephone number if they required immediate contact. Patients had few problems assessing and using the site.

**Fig. 3:**
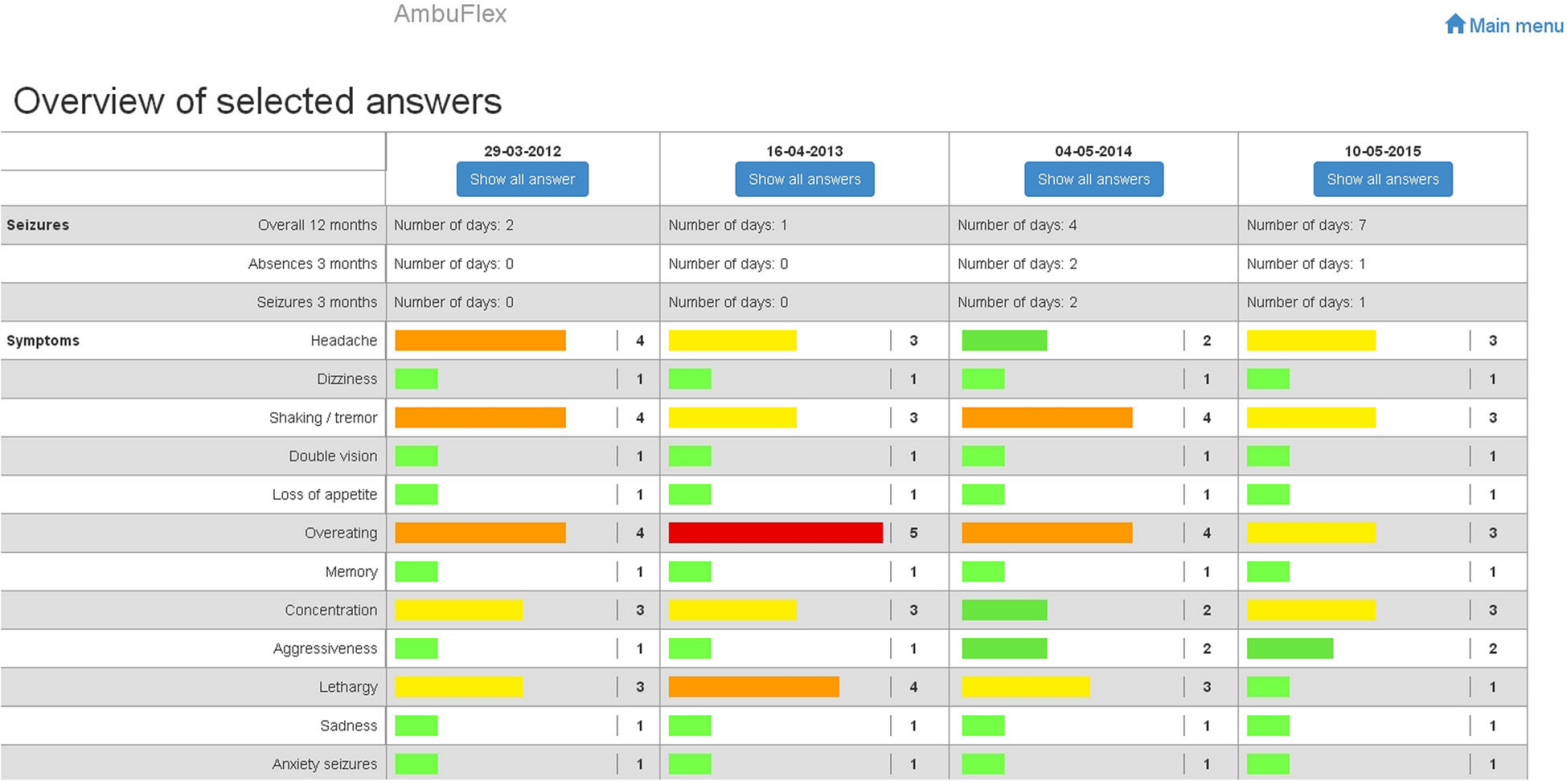
PRO response overview customised to outpatients with epilepsy. A red or orange bar indicates a self reported problem, a yellow bar some problem, and green bar indicates no problems. Note: Labels were translated from Danish
- Info: This element includes information about the open access approach including detailed information about the purpose and how to use the website. In addition, there is information about the questionnaire and why it is important to gather information about the included aspects, e.g., seizures, alcohol, pregnancy, sexuality, etc. The information provided is compiled by clinicians from the epilepsy clinics at Aarhus University Hospital and is based on disease-specific guidelines and information from the Danish Epilepsy Association.
- Contact: Patients are asked to contact the epilepsy clinic by telephone in the event of a pressing need of attention. This element contains contact information (telephone number, email and mail addresses) to the epilepsy clinic. The emergency service is always open for those in acute need for help, for example, if the patient gets a seizure.

The website consists of four core elements:

- Answer questionnaire: Here, patients can answer the epilepsy questionnaire when they need to get in contact with the clinic. The questionnaire is the same as in standard telePRO. When the patient has completed the questionnaire, the response is automatically sent as a red request to the PRO alert list at the epilepsy clinic. The clinician assesses the response and contacts the patient as soon as possible. The clinic has reserved appointments in their booking system to ensure that patients get a quick appointment. As a “safety net”, patients have to answer the questionnaire before twice the fixed interval has elapsed. For example, if the patient is referred with a 12-month interval, the patient has to respond within two years. If not, the patient is automatically sent a questionnaire, given a red status, and is contacted by a clinician.
- Previous answers: In this element, all of the patient’s previous questionnaire responses are available. Patients have access to a PRO overview interface and specific and detailed questionnaire responses in the same manner as the clinicians. The overview interface is shown in Fig. 3. It is customised to monitor selected PRO data and to illustrate changes in health status over time. Colour codes indicate the severity of the symptoms reported by the patient. A red or orange bar indicates a self-reported problem, a yellow bar some problem, and green bar indicates no problems.

### Randomisation

Pre-randomisation designs prevent change in behaviour in the control group because of disappointment about the allocation [19]. Eligible standard telePRO participants will be pre-randomised to standard telePRO follow-up (no change) or open access telePRO follow-up. Control as well as intervention participants receive the baseline questionnaire together with the fixed PRO questionnaire. The clinicians respond to the fixed PROs as usual and will not have access to the baseline questionnaire. Control participants will continue with fixed interval questionnaires and no change will be undertaken. Intervention participants will receive detailed information about the open access approach two weeks after a clinician’s response to the fixed PRO questionnaire. The study coordinator will forward written information to the included intervention participants. Individuals who not agree to participate will continue with standard telePRO follow-up. Due to the nature of the intervention neither patients nor clinicians can be blinded to allocation. The randomisation is performed with an algorithm developed as part of the WestChronic software [12]. The allocation ratio open access/standard is 0.55/0.45. This ratio was selected to account for an expected number of patients in the open access arm who do not wish to participate.

### Study timeline

**Fig. 4:**
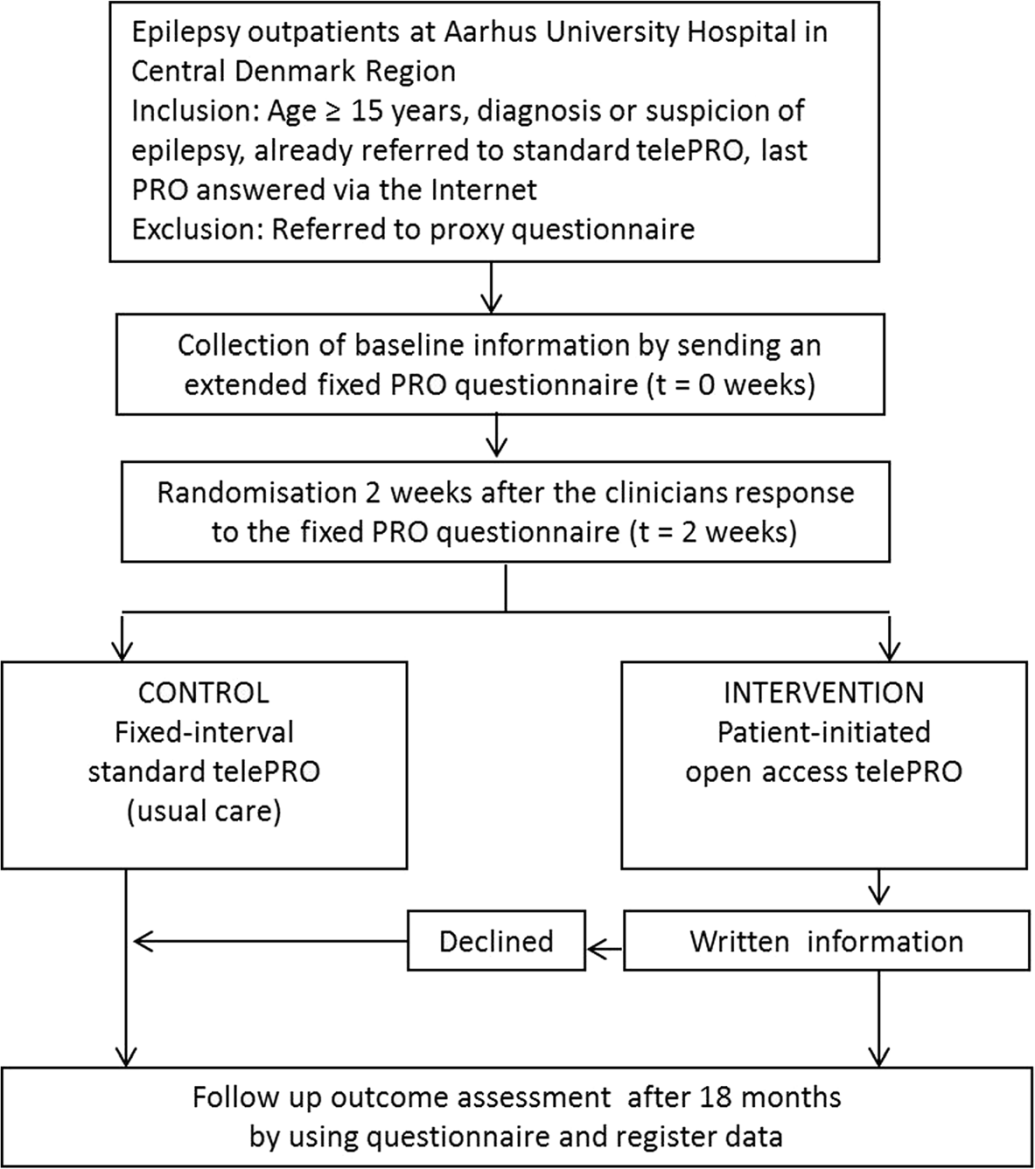
Flowchart following patients from inclusion to final data collection

Inclusion and randomisation with baseline assessments will take place from January 2016. Follow-up assessment will take place 18 months after randomisation. Baseline and follow-up assessments are shown in Table 1. Figure 4 presents the inclusion of patients and the stages in the study.

**Table 1:** Primary and secondary outcomes, data sources, and timeline for measurements

Outcomes	Data sources	Measurement/month
Resource utilisation		
1. Number of contacts	The Hospital Business Intelligence Register, Central Denmark Region	0–18
Quality of care		
2. Well-being	WHO-Five Well-being Index (WHO-5)	0, 18
3. General health	Item from The Short Form Health Survey (SF-36)	0, 18
4. Mortality	The Hospital Business Intelligence Register, Central Denmark Region	0–18
5. Number of seizures	Item from the epilepsy questionnaire, Central Denmark Region	0, 18
6. Treatment side effects	Item from the epilepsy questionnaire, Central Denmark Region	0, 18
Patient perspective ^a^		
7. Health literacy	The Health Literacy Questionnaire (HLQ) sub scale 4, 6 and 9	0, 18
8. Self-efficacy	General Self-Efficacy scale (GSE)	0, 18
9. Patient activation	Items from Patient Activation Measure (PAM)	0, 18
10. Confidence, safety, and satisfaction	Items from a PREM questionnaire, Danish Cancer Society	0, 18

^a^ The patient perspective is primarily explored by qualitative methods in a complementary PhD study

### Outcomes

The effects of patient-initiated follow-up (open access telePRO) will be evaluated with regard to three different aspects: resource utilisation, quality of care, and the patient perspective. Resource utilisation will constitute the primary outcome, measured by number of contacts. Quality of care and the patient perspective constitute the secondary outcomes. Quality of care includes pivotal clinical quality measures (mortality, seizure, and treatment side effects) as well as more general patient-oriented quality measures (well-being and general health). The patient perspective includes measures related to self-management, such as health literacy, self-efficacy, and patient activation. Measures of confidence, safety, and satisfaction will be used to describe patient experiences. The patient perspective is primarily explored by qualitative methods in a complementary PhD study. An overview of primary and secondary outcomes, data sources, and measurement timeline is shown in Table 1.

### Primary outcome

#### Resource utilisation

Number of contacts includes all contacts with the outpatient clinic in the study follow-up period, including face-to-face consultations with a physician, face-to-face consultations with a nurse, and telephone consultations. In addition, other health care contacts will be gathered, e.g., epilepsy-related emergency room visits and hospitalisations as well as hospitalisation related to co-morbidity. Data will be gathered from the Hospital Business Intelligence Register in Central Denmark Region.

### Secondary outcomes

#### Quality of care

Patients’ well-being will be measured by using the Danish version of WHO-Five Well-being Index (WHO-5). WHO-5 was developed by the World Health Organisation for the assessment of well-being among patients with diabetes [20]. WHO-5 consists of five positively worded items reflecting present mental well-being within the previous two weeks. Items are rated on a 6-point scale ranging from 5 “all of the time” to 0 “at no time”. The instrument has demonstrated sufficient psychometric properties in a wide range of chronic conditions [20, 21]. Patients’ general health will be measured by using one item from the Danish version of The Short Form Health Survey (SF-36); *“In general, would you say your health is: excellent, very good, good, fair, or poor”* [22, 23]. The validity and reliability of this item are well documented [24]. Data on mortality will be gathered from the Hospital Business Intelligence Register in Central Denmark Region. Finally, number of seizures and treatment side effects will be collected from ad hoc items in the epilepsy questionnaire used at epilepsy clinics in Central Denmark Region. The validity and reliability of these items have not yet been documented.

### Patient perspective

Health literacy will be measured by using the Danish version of Health Literacy Questionnaire (HLQ) [25, 26]. HLQ was developed to measure a wide range of health literacy needs of people in the community. The HLQ includes nine conceptually subscales with a total of 44 items containing five scales with agree/disagree response options and four scales with difficulties in perform tasks response options. The HLQ has well-documented psychometric properties [26]. In this study, the HLQ subscales 4, 6, and 9 will be used; *4. Social support for health, 6. Ability to actively engage with healthcare providers, 9. Understand health information well enough to know what to do.* Self-efficacy will be measured by using the Danish version of General Self-Efficacy Scale (GSE) [27, 28]. GSE was designed to assess optimistic self-belief to cope with difficult demands in life [27, 28]. GSE includes ten items with a response range from 1 “not at all true” to 4 “exactly true”. The GSE scale has been used in a range of research projects in different countries and populations, where it typically yielded sufficient psychometric properties [29]. Patient activation will be measured by two ad hoc items developed with inspiration from the Danish version of the Patient Activation Measure (PAM) [30]. Confidence, safety, and satisfaction will be measured by using ad hoc items developed with inspiration from a Danish PREM (patient-reported experience measure) questionnaire from the Danish Cancer Society.

In addition, the patient perspective will be explored in a complementary qualitative PhD study. The primary aim of this study is to explore the mechanisms of actions related to standard telePRO and open access telePRO. Interpretive description (ID) will be used as the research approach [31]. Patients’ experiences with telePRO will be explored in individual interviews and participant observations in outpatient clinics. The target group for participation is patients with epilepsy, referred to standard telePRO or open access telePRO follow-up in the three neurological departments in Central Denmark Region.

### Other measurements

Demographic information such as sex, age, education, marital status, and duration of epilepsy diagnosis will be obtained from baseline questionnaires.

### Sample size

Statistical power was estimated for the primary outcome number of contacts. Based on literature review [32] the number of consultations (n) and standard deviation (SD) was; *n* = 4.64, (SD = 2.38) in conventional follow-up and *n* = 4.12, (SD = 3.41) in open access follow-up. We expect at least a difference of one contact between the groups. Given a statistical power of 90%, *p*-value 0.05, and allocation ratio 0.8, we will need a sample size of 172 patients in the standard telePRO group and 214 patients in the open access telePRO group. To account for attrition and loss to follow-up, we will recruit a total of approximately 500 participants. For qualitative data, a purposeful sample of at least five participants from each group will be interviewed.

### Analyses

Descriptive statistics will be used to describe differences in the baseline characteristics of participating patients in the two arms of the trial. Statistical analysis will be intention to treat, whereby all randomised participants will be included in the analysis according to their randomised allocation. The primary outcome, total *number of contacts* in the two arms*,* will be analysed using a sample t-test. If the distribution of data is skewed, we will use medians and nonparametric tests. For secondary outcomes, a chi-square test or logistic regression will be used for dichotomous outcome data and sample *t*-test or multiple linear regression analysis will be used for continuous outcome data. Non-parametric tests will be used if continuous data are not normally distributed. Demographics covariates (sex, age, education, marital status, and epilepsy diagnosis duration) will be included in the per- protocol analysis.

For qualitative data, ID will be employed as the overriding research approach. ID is an inductive research strategy in which constant comparative method with concurrent data collection and analysis is utilised to gain a deeper insight and understanding of human experiences within their natural context. The result is a comprehensive interpretation, potentially a *model of explanation* of the phenomenon under study, which can provide clinical practice with a research-based choice of action [31]. ID is considered appropriate in the present study because the approach is suited for exploration of specific clinical issues, in this case how patients with epilepsy experience standard and open access telePRO follow-up.

### Ethics

The risks to participants are considered to be minimal as all eligible participants are referred to standard telePRO follow-up by clinicians at the epilepsy clinic. As a “safety net” to ensure that no patients are lost in the open access arm, the patients have to answer the epilepsy questionnaire before twice the fixed interval has elapsed. If lack of response the patient is reallocated into standard telePRO with a red status and a clinician will contact the patient. Furthermore, all patients are informed to call the clinic in pressing need of attention.

The Danish Data Protection Agency has accepted the study. In addition, the Danish research ethics committee in Central Denmark Region was contacted and has stated that approval from the committee is not necessary for this present study. Therefore, written informed consent was not obtained from the participants. Prior to study participation patients in the intervention group receive written information about the study. Study participation is entirely voluntary and participants are informed they can withdraw from the study at any time without affecting future care. In the qualitative complementary PhD study, the participants gave written informed consent prior to enrolment, and the study was approved by the Danish Data Protection Agency.

### Data security

All data activities in the study are documented and stored in the WestChronic web-system [12]. The system is physically located in Central Denmark Regions Server Park behind the firewall and Threat Management Gateway. Regular backup is performed weekly. All data transactions fulfill conditions established by the Danish Data Protection Agency.

## Discussion

During the last decade, the use of PRO in clinical practice has become increasingly common, and to our knowledge, AmbuFlex is the first generic PRO system that uses PRO as the basis for outpatient follow-up [13]. The focus of this trial will be to evaluate the effect of a patient-initiated open access telePRO intervention compared to standard telePRO with respect to resource utilisation, quality of care, and the patient perspective. Ideally, we would have preferred to compare the two arms (standard and open access) with conventional follow-up with pre-booked outpatient visits to the clinic. However, this was not possible because the epilepsy clinics in Central Denmark Region have used standard telePRO follow-up since 2012. Thus, we will compare two rather similar outpatient follow-up activities, which will probably result in only small differences in effect between the groups. Evaluation of the effect must be done using reliable, valid, and clinically meaningful measures. This study includes outcome measures based on recommendations from clinical experts, researchers, and the literature [33].

Loss to follow-up is one of the main concerns in randomised controlled studies [34]. Loss to follow-up in this study is related to the open access group of patients since study participation in the open access arm is entirely voluntary, and participants can choose to continue with standard telePRO follow-up. Loss of statistical efficiency can be overcome by increased the number of participants in the study [19]. We have taken this into consideration and will include 10% more patients in the open access telePRO arm. In addition, we will recruit a larger number of participants than the minimum sample size calculation indicated.

Only web-responders will be included in the open access arm, and the results may therefore be generalizable only to this subgroup of epilepsy patients. These patients may differ with respect to education, age, and use of new technologies compared to the entire group of epilepsy patients. In another study in progress, the aim is to examine determinants for referral to telePRO follow-up. Data from this study can be used to compare the study population with the entire group of patients with epilepsy.

Another potential challenge may be how individuals in the intervention group will use the “My Epilepsy” website. Some patients are better able to decide themselves when they need to contact the clinic, while others are more reserved and afraid to be a nuisance. Several patients that have used standard telePRO have pointed out the benefit of getting a fixed questionnaire once a year. They do not believe they would remember to answer if they had to do it on their own. This could signify that even though they may not feel the need for a clinical appointment, but do feel a form of security in answering the fixed interval questionnaire. This will be taken into consideration in the study, since all patients in the intervention group will receive a questionnaire if they do not respond within two times the referred interval, for example, within 24 months if they are assigned a 12-month questionnaire interval. Another concern could be that patients in the open access group could choose to make a call instead of answering the questionnaire when they need to get in contact with the clinic. If they behave in this way, the benefit of using PRO in clinical practice will be reduced.

Standard telePRO has been well integrated into clinical practice in three epilepsy clinics in Central Denmark Region since 2012. A new patient-initiated approach has been developed that may result in potential benefits in terms of the patient perspective and resource utilisation. The potential benefits as well as possible drawbacks need to be evaluated. We have decided to combine qualitative and quantitative research methods in two parallel PhD studies. The intention of the complementary qualitative PhD study is to further explain the findings from the randomised study by providing a description of the various ways in which telePRO is manifested and an interpretation of the underlying mechanisms of action. The two studies will complement each other and contribute with important research-based knowledge to guide future telePRO interventions in relation to effect on resource utilisation, quality of care, and the patient perspective.

### Trial status

On going.

## Additional file

Additional file 1: SPIRIT 2013 Checklist: Recommended items to address in a clinical trial protocol and related documents*. (DOC 122 kb)

## Abbreviations

GSE: General Self-Efficacy scale
HLQ: Health literacy questionnaire
ID: Interpretive description
PAM: Patient activation measure
PREM: Patient-reported experience measure
PRO: Patient-reported outcome
SD: Standard deviation
SF-36: Short form health survey
WHO-5: WHO-Five well-being index
